# Transgenerational inheritance of adrenal steroidogenesis inhibition induced by prenatal dexamethasone exposure and its intrauterine mechanism

**DOI:** 10.1186/s12964-023-01303-0

**Published:** 2023-10-18

**Authors:** Zheng He, Jinzhi Zhang, Yawen Chen, Can Ai, Xiaohan Gong, Dan Xu, Hui Wang

**Affiliations:** 1 Department of Pharmacology, Wuhan University School of Basic Medical Sciences, Wuhan, 430071 Hubei Province China; 2 Department of Pharmacy, Tongji Hospital, Tongji Medical College, Huazhong University of Science and Technology, Wuhan, 430030 China; 3 Hubei Provincial Key Laboratory of Developmentally Originated Disorder, Wuhan, 430071 China

**Keywords:** Prenatal dexamethasone exposure, H19, Let-7 axis, Adrenal steroidogenesis, Maternal multigenerational inheritance, Oocytes

## Abstract

**Background:**

Adrenal gland is the synthesis and secretion organ of glucocorticoid, which is crucial to fetal development and postnatal fate. Recently, we found that prenatal dexamethasone exposure (PDE) could cause adrenal dysfunction in offspring rats, but its multigenerational genetic effects and related mechanisms have not been reported.

**Methods:**

The PDE rat model was established, and female filial generation 1 (F1) rats mate with wild males to produce the F2, the same way for the F3. Three generation rats were sacrificed for the related detection. SW-13 cells were used to clarify the epigenetic molecular mechanism.

**Results:**

This study confirmed that PDE could activate fetal adrenal glucocorticoid receptor (*GR*). The activated *GR*, on the one hand, up-regulated *Let-7b* (in human cells) to inhibit steroidogenic acute regulatory protein (*StAR*) expression directly; on the other hand, down-regulated CCCTC binding factor (*CTCF*) and up-regulated DNA methyltransferase 3a/3b (*Dnmt3a/3b*), resulting in *H19* hypermethylation and low expression. The decreased interaction of *H19* and *let-7* can further inhibit adrenal steroidogenesis. Additionally, oocytes transmitted the expression change of *H19/let-7c* axis to the next generation rats. Due to its genetic stability, F2 generation oocytes indirectly exposed to dexamethasone also inhibited *H19* expression, which could be inherited to the F3 generation.

**Conclusions:**

This cascade effect of *CTCF/H19/Let-7c* ultimately resulted in the transgenerational inheritance of adrenal steroidogenesis inhibition of PDE offspring. This study deepens the understanding of the intrauterine origin of adrenal developmental toxicity, and it will provide evidence for the systematic analysis of the transgenerational inheritance effect of acquired traits induced by PDE.

Video Abstract

**Supplementary Information:**

The online version contains supplementary material available at 10.1186/s12964-023-01303-0.

## Introduction

In the 1990s, David Barker first proposed the increased incidence of hypertension and diabetes in adulthood with low birth weight and the hypothesis of “fetal-originated diseases” based on epidemiological investigations. Later, scholars put forward a new concept of the origin of human diseases, called “Developmental Origins of Health and Disease (DOHaD)” theory based on the results of a large number of evidence-based studies [1], which suggested that adverse environmental factors in early life may cause fetal dysplasia and permanently program the occurrence and development of adult diseases [2, 3], especially about a variety of metabolic diseases [4]. As an essential endocrine organ of hypothalamic–pituitary–adrenal (HPA) axis, the adrenal gland is mainly responsible for synthesizing and secreting glucocorticoids. Studies have confirmed that intrauterine basal glucocorticoid levels mainly come from the mother, which is the key to promoting fetal tissue morphology and function development [5–7]. After birth, glucocorticoids are considered the most sensitive hormone to induce insulin resistance [8, 9].

Dexamethasone is a synthetic glucocorticoid, which is widely used in pregnant women at risk of premature delivery to promote lung maturation of premature infants and prevent the occurrence of neonatal respiratory distress [10, 11]. Many studies have shown that prenatal dexamethasone exposure (PDE) could cause intrauterine growth retardation (IUGR) in fetuses and increase the susceptibility to many chronic diseases in adulthood, such as osteoporosis, glomerular sclerosis, testis, and ovarian dysfunction [12–15]. Studies reported prenatal dexamethasone treatment could significantly reduce the fetal adrenal volume and steroid synthesis [16, 17]. Our latest study found that PDE could induce IUGR and inhibit adrenal steroidogenesis in male offspring rats [18], while abnormal secretion of glucocorticoids is the main factors of susceptibility to metabolic diseases in adult offspring [19]. These findings suggested that the susceptibility to various chronic diseases in adulthood in IUGR offspring is related to abnormal adrenal development. More and more evidence indicated that the acquired phenotypes of offspring caused by the adverse environment encountered by parents could be inherited for multiple generations [20–23]. Our previous work found that the effects of PDE on the long bone and ovary in offspring rats could be inherited through maternal line to the F2 or even the F3 [13, 24]. These studies suggested that adrenal developmental toxicity in offspring induced by PDE may also have multigenerational inheritance effects.

Since most environmental factors can’t cause heritable DNA sequence changes, epigenetic modification abnormalities are considered to be the primary mechanism of the multigenerational inheritance of acquired phenotypes [25], and germ cells are considered to be an important medium for the transmission of epigenetic information between parents and offspring [26]. As one of the maternal imprinted genes, H19 is responsible for transmitting maternal imprinting state phenotype to the progeny [27], and oocyte is an important medium for the parental transmission of H19 epigenetic information. Research shows that the differential methylation region (DMR) in the H19 promoter region determines its imprinting state and expression [27], which is regulated by CCCTC binding factor (CTCF) and DNA methyltransferases (Dnmts) [28, 29]. Studies have shown that abnormal expression of H19 during pregnancy could lead to IUGR [30]. It is known that let-7 family is involved in various regulatory processes of body development, and there are differences in let-7 among different species (such as let-7b in humans and let-7c in rats). H19 could negatively regulate the activity of the let-7 family through “molecular sponge” action, thereby involved in the regulation of transcription expression of its target genes [31, 32]. Steroidogenic acute regulatory protein (StAR) is a critical regulator protein, which is essential for adrenal glucocorticoids synthesis and developmental stability [33–35]. Literature suggested that miRNA let-7 family regulates StAR expression in human granulosa cells [36]. Therefore, the H19/let-7 pathway may mediate the inhibition of adrenal steroidogenesis and its maternal multigenerational inheritance effects induced by PDE.

In this study, we intended to observe the changes of adrenal steroidogenic function and its maternal multigenerational genetic effects in PDE offspring rats. Further, we would elucidate the epigenetic regulatory mechanism of the change in adrenal steroidogenic function induced by dexamethasone in vivo* and *in vitro. Finally, we tried to confirm the mechanisms of intergenerational inheritance mediated by oocyte transmission. This study provided an experimental basis for explaining the adrenal developmental toxicity, and also provides new ideas for exploring the multigenerational inheritance effects of fetal-originated diseases.

## Materials and methods

### Materials

Dexamethasone was obtained from Shuanghe Pharmaceutical Company (Wuhan, China). Human chorionic gonadotropin (hCG) was provided by ProSpec-Tany TechnoGene Ltd (Ness-Ziona, Israel). Pregnant mare serum gonadotropin (PMSG) was obtained from Head Biotechnology Co., Ltd (Beijing, China). Rat corticosterone enzyme-linked immunosorbent assay (ELISA) kit was provided by Assay-pro LLC (Saint Charles, Missouri, USA). Glucocorticoid receptor (GR) antagonist mifepristone (RU486) was purchased from Gene Pharma (Shanghai, China). The CTCF and H19 plasmid, GR and Dnmt3a siRNA were obtained from Suzhou Gene Pharma Co., Ltd (Suzhou, China), and let-7b inhibitor was purchased from Guangzhou Ribo Bio Co., Ltd (Guangzhou, China). Primary antibodies, such as anti-GR, Dnmt3a (ChIP, ab2850), GR (ChIP, ab2768), CTCF (ChIP, ab188408), and StAR were purchased from Abcam plc (Cambridge, Cambridge shire, UK). Anti-glyceraldehyde-3-phosphate dehydrogenase (GAPDH) was obtained from ABclonal Science, Inc (Woburn, Massachusetts, USA), and anti-CTCF was purchased from Cell Signaling Technology, Co (Beverly, MA).

### Animals and treatments

SPF Wistar rats (weights of 200–240 g for females and 260–300 g for males, No. 2012–2014, certification number: 42000600002258) were obtained from the Experimental Center of Hubei Medical Scientific Academy (No. 2009–2014, Hubei, China). Animal experiments were performed in the Animal Experimental Center of Wuhan University (Wuhan, China), which was accredited by the Association for Assessment and Accreditation of Laboratory Animal Care International (AAALAC International). The Animal Experiment Ethics Committee of Wuhan University Medical College approved the program (permit number: 201709). All animal testing procedures were performed following the Guidelines of the Chinese Animal Welfare Committee on experimental animals.

All rats were fed at standard conditions (room temperature: 18–22 ℃; humidity: 40%-60%; light cycle: 12 h light–dark cycle) for one week, and male and female rats were placed in a ratio of 1:2 for mating at 6:00 pm. The vaginal smear was examined the following day, and the day when the sperm was observed was the gestational day (GD) 0. From GD9 to GD20, pregnant rats in the experimental group were given 0.2 mg/kg·d of dexamethasone subcutaneously, and the control group were given an equal volume of normal saline. On GD20, part of the pregnant rats was euthanized under 2% isoflurane anesthesia. Pregnant rats with litter sizes of 8 to 14 qualified. IUGR was diagnosed when the bodyweight of an animal was two standard deviations less than the mean bodyweight of the control group. Fetal blood samples were collected, and serum was isolated. Some fetal adrenals were randomly selected and fixed in the 4% paraformaldehyde solution overnight, then dehydrated in alcohol and embedded in paraffin for later use. The other fetal adrenals were immediately frozen in liquid nitrogen and stored at − 80 ℃ for further analysis.

**Fig. 1:**
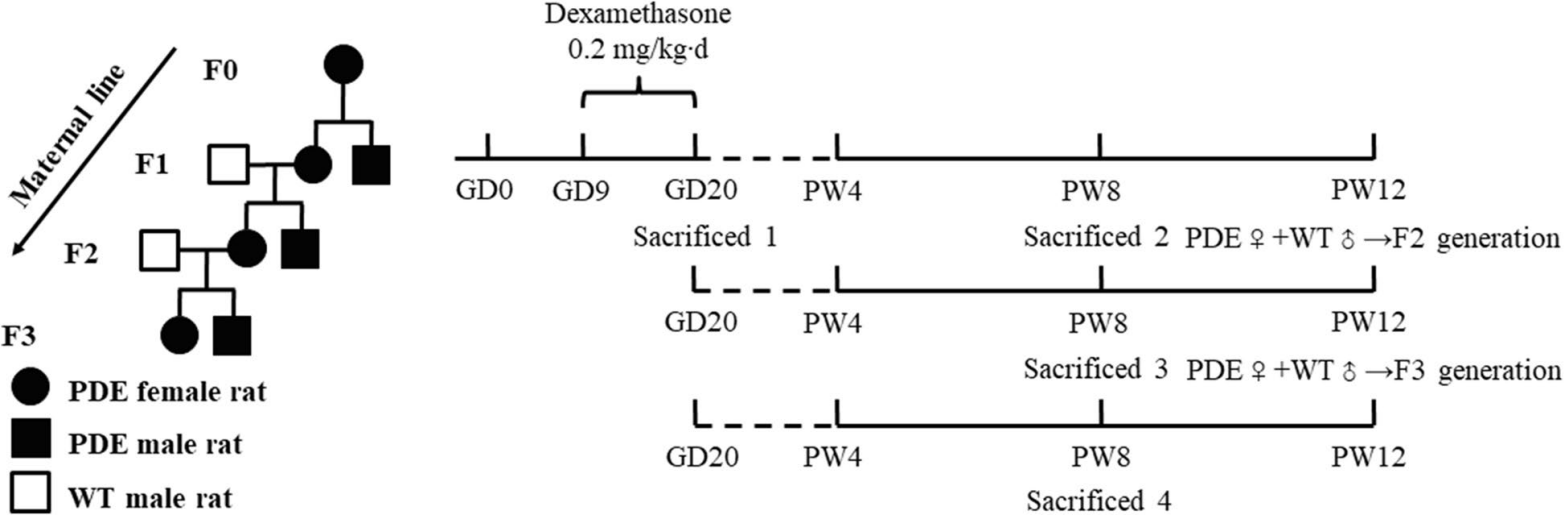
Prenatal dexamethasone exposure (PDE) model and multigenerational phenotype via maternal-line. GD: gestational day; PW: postnatal week; WT: wild type

The remaining pregnant rats were raised to normal delivery and produced the first generation (F1). It is necessary to balance the litter size (*i.e.*, nutritional balance) of each pregnant rat to avoid the individual difference caused by the postnatal rapid development period. So, we adjusted the litter size to 12 neonatal rats per litter (male/female ratio was approximately 1:1) after a natural delivery. After weaning, all female F1 offspring were maintained on standard laboratory chow ad libitum. Part of the F1 offspring was sacrificed with 2% isoflurane anesthesia; serum and adrenals were collected at postnatal week (PW) 8. Part of the female offspring was treated with PMSG (40 IU) and human chorionic gonadotrophin (hCG, 40 IU) to obtain oocytes at PW12. The rest female offspring were mated with wild-type (WT) male rats at PW12 to produce the second generation of offspring (F2). In the same way, F2 rats were collected serum and adrenals at PW8 and obtained oocytes or consecutively produced the third-generation (F3) at PW12. The F3 rats used the same protocol as the F1/F2 to collect blood and adrenals at PW8. The mating method was shown briefly as below (Fig. 1).

### NCI-H295R cell culture and treatments

The human adrenocortical cell line (NCI-H295R) was provided by Shanghai Zishi Biotechnology Co., Ltd. (Shanghai, China). The cells were cultured in a 5% CO_2_ humidified incubator at 37 ℃. The standard medium for SW-13 cells was DMEM supplemented with 2% FBS, 0.1% SIT, and penicillin/streptomycin. According to our previous data, serum dexamethasone levels of maternal, male and female fetal rats were 0.394 μg/mL (846 nM), 0.11 μg/mL (236 nM) [12] and 0.124 μg/mL (267 nM), respectively [13]. Based on these, dexamethasone at different concentrations (20, 100, 500 2500 nM) was applied to the NCI-H295R cells for 12, 24 and 48 h. Mifepristone (RU486) is a glucocorticoid receptor (GR) antagonist. RU486 can competitively bind GR and block glucocorticoids to exert biological effects on the cells. The concentration of the GR siRNA, let-7b inhibitor, CTCF plasmid, DNNMT3a siRNA, H19 plasmid and RU486 are treated according to the manufacturer’s protocol. So, we also transfected the cells with GR siRNA (30 nM), let-7b inhibitor (50 nM), CTCF plasmid (1000 ng/mL), DNNMT3a siRNA (30 nM), H19 plasmid (1000 ng/mL) or RU486 (2500 nM) and treated with 500 nM dexamethasone for 24 h, then collected the cells for further analysis.

### Analysis of serum corticosterone concentration

Serum corticosterone concentration was measured with 50 μL serum using an ELISA kit according to the manufacturer's protocol. The detection limit of corticosterone was 0.39 ng/mL. As defined by the manufacturer, the intra-assay and inter-assay coefficients of variation for corticosterone were 5.0 and 7.2%, and the cross-reactivity for the corticosterone ELISA was 2%.

### Immunohistochemical examination

Serial sections of 5 μm thickness were taken from the mid-portion of each adrenal, dewaxed, and washed in PBS. After antigen retrieval, adrenal sections were blocked in 5% blocking serum for 30 min at room temperature and incubated overnight at 4 ℃ with primary antibodies (anti-StAR, 1:500 dilution, anti-CTCF, 1:100 dilution). After primary antibody incubation, the sections were incubated with a biotinylated secondary antibody and then with an avidin-biotinylated horseradish peroxidase complex solution according to the manufacturer’s directions. Immunohistochemical analysis was performed using a DAB staining kit to determine the expressional levels of StAR and CTCF proteins in the adrenals. All images were analyzed using Image-Pro Plus software (version 6.1, Media Cybernetics, Silver Spring, USA). The staining intensity was determined by measuring the mean optical density in five random fields for each section.

### Total RNA extraction, reverse transcription, and RT-qPCR

Adrenal gland tissue or NCI-H295R cells were homogenized in Trizol reagent. The total RNA and miRNA were extracted according to the manufacturer’s protocols. The concentration and purity of the isolated total RNA and miRNA were determined by spectrophotometer (Nano Drop 2000C, Thermo). The total RNA concentration was adjusted to 1000 ng/μL, and miRNA concentration was adjusted to 200 ng/μL. Total RNA and miRNA were separately converted to cDNA according to the protocol of the reverse transcription kit. Primer sequences of the analysis genes are shown in Table 1. RT-qPCR was performed using the ABI Step One RT-PCR thermal cycler (ABI Stepone, NY, USA) in a 10 μL reaction mixture. The mRNA level of GAPDH and miRNA level of U6 were measured as a quantitative control.

**Table 1:** Oligonucleotide primers in real-time quantitative PCR

Species	Genes	Forward primer	Reverse primer
Rat	StAR	GGGAGATGCCTGAGCAAAGC	GCTGGCGAACTCTATCTGGGT
Rat	GR	CACCCATGACCCTGTCAGTC	AAAGCCTCCCTCTGCTAACC
Rat	CTCF	ATGATCCCAACTTTGTCCCC	CCTCCATTTTCCCCTTCTACAC
Rat	H19	CCTCAAGATGAAAGAAATGGTGCTA	TCAGAACGAGACGGACTTAAAGAA
Rat	Dnmt3a	GGTCATGTGGTTCGGAGATG	AGGACTTCGTAGATGGCTTTG
Rat	Dnmt3b	TGGTAGGAGATGGAGATGGTG	AGATACTGTTGCTGTTTCGGG
Rat	GAPDH	GCAAGTTCAATGGCACAG	GCCAGTAGACTCCACGACA
Human	StAR	GGCATCCTTAGCAACCAAGA	TCTCCTTGACATTGGGGTTC
Human	GR	CCCGTTGGTTCCGAAAATTG	AGCTTACATCTGGTCTCATGC
Human	CTCF	CTGTTCCTGTGACTGTACCTG	AAAGGTAGGGTGTGGCATATC
Human	H19	GCACCTTGGACATCTGGAGT	TTCTTTCCAGCCCTAGCTCA
Human	Dnmt3a	ATGGGCGTTAGTGACAAGAG	TCACAGTGGATGCCAACG
Human	Dnmt3b	CCCATTCGAGTCCTGTCATTG	TTGATATTCCCCTCGTGCTTC
Human	GAPDH	ACATCGCTCAGACACCATG	TGTAGTTGAGGTCAATGAAGGG

*StAR* steroidogenic acute regulatory protein, *GR* glucocorticoid receptor, *CTCF* CCCTC-binding factor, *Dnmt3a* DNA methyltransferase 3 alpha, *Dnmt3b* DNA methyltransferase 3 beta, *GAPDH* glyceraldehyde-3-phosphate dehydrogenase

### Bisulfite sequencing PCR (BSP)

Genomic DNA was extracted from adrenal tissue or SW-13 cells using the DNA isolation kit (Tiangen Co., Beijing, China). Genomic DNA (1 μg) was analyzed using the EZ DNA Methylation kit (Zymo Research Co., CA, USA) and treated with bisulfite. The amplified product was purified by the purification kit (Tiangen Co., Beijing, China) and then inoculated into the pUC18-T vector plasmid (Sangon Biotech Co., Ltd., Shanghai, China). Ten clones of each sample were selected for sequencing analysis. The BSP primers used for PCR amplification are shown in Table 2.

**Table 2:** Oligonucleotide primers in bisulfite sequencing PCR

Species	Genes	Forward primer	Reverse primer
Rat	H19	CAGGTTCAACAAAGGGGTCAGG	AGCCATAAAAAGAGGCTGAGATTCAAC
Human	H19	TTGGTAGGTATAGAAATTGGGG	AAACCATAACACTAAAACCCTCA

### Chromatin immunoprecipitation (ChIP) assay

ChIP assays were performed on NCI-H295R cells to evaluate the levels of CTCF and Dnmt3a binding of the H19 ICR, and GR binding of miRNA let-7 promoter according to a modified version of the manufacturer’s protocol. The samples were cross-linked with 1% formaldehyde on a rocker for 10 min, followed by the addition of 125 mM glycine to stop the reaction. Samples were washed by ice-cold PBS and disaggregated by using a Dounce homogenizer in lysis buffer. The suspension was then centrifuged at 1500 × g, 4 ℃ for 60 s, followed by resuspension and sonication (TIMER 2 s, PULSE 1 s, AMPL 30%, 5 min) using SONICS Vibra-Cell TM (Cole-Parmer Instruments, Vernon Hills, IL) to obtain 200 to 1000 bp fragments. The sonicated samples were then incubated with protein G magnetic beads (Millipore, 16–157) and divided into three aliquots: the first was used for input DNA. The other two were used for immunoprecipitation with anti-CTCF, anti-GR, anti-Dnmt3a antibody and IgG (ab171870, Abcam) at 4 ℃ overnight on a rocker, followed by incubation with proteinase K (200 μg/mL) at 65 ℃ overnight. DNA was isolated from each immune-precipitate and subjected to RT-qPCR analysis. The primers used are shown in Table 3.

**Table 3:** Oligonucleotide primers in real-time quantitative PCR

Species	Genes	Forward primer	Reverse primer
Human	H19	TCAGACACCGAAATCAACGA	TCAGCAGGGATGCGATGTA
Human	miR-let-7b	TCATGCTACACTCTGTCCCA	TTGAGGGAAGTGGCCTCTAG

### Western blot analysis

NCI-H295R cells and ground adrenal tissue were lysed in RIPA lysis buffer containing protease inhibitors for 30 min at 4 ℃. The cell lysates were then centrifuged at 12,000 × g for 10 min at 4 ℃. The supernatant was harvested, and the protein concentration was determined using a BCA protein assay kit. Aliquots of lysate were mixed with 5 × loading buffer containing 2-mercaptoethanol and boiled at 100 ℃ for 5 min, then loaded onto 10% SDS polyacrylamide gels and electrophoresis (SDS-PAGE) was performed. After SDS-PAGE separation, proteins were transferred to a PVDF membrane (Millipore, Billerica, MA, USA) using a BioRad gel system. After blocking at room temperature for 2 h with 5% non-fat milk in Tris-buffered saline with Tween (TBST, 25 mM Tris–HCl, 50 mM NaCl, 0.05% Tween-20), the membranes were incubated with the diluted primary antibodies (anti-GR, 1:1000 dilution; anti-StAR, 1:500 dilution; anti-CTCF, 1:1000 dilution; anti-GAPDH, 1:5000 dilution) overnight at 4 ℃. Following extensive washing, the membrane was incubated in the dark for 1 h with a fluorescent secondary antibody at 1:10,000 dilution in a blocking solution containing 0.1% Tween-20. The fluorescent secondary antibodies were Alexa Fluor 680 goat anti-mouse molecular probes and Alexa Fluor 800CW goat anti-rabbit molecular probes (Rockland Immunochemical, PA). Specific bands were detected using the electro-chemi-luminescence (ECL) assay.

### Statistical analysis

SPSS19 (SPSS Science Inc., Chicago, Illinois) and Prism 6.0 (GraphPad Software, La Jolla, CA, USA) were used for data analysis. All presented measurement data except IUGR rates were expressed as mean ± S.E.M. The *t-*test and One-way ANOVA were used as appropriate. The mean bodyweight of each litter was used for statistical analyses, and the IUGR rate was arcsine square-root transformed before* t*-test evaluations. Statistical significance was defined as *p* < 0.05.

## Results

### Effects of PDE on adrenal steroidogenesis in female fetal rats and its molecular mechanism

#### Effects of PDE on adrenal steroidogenesis and related H19/let-7c axis in fetal rats

**Fig. 2:**
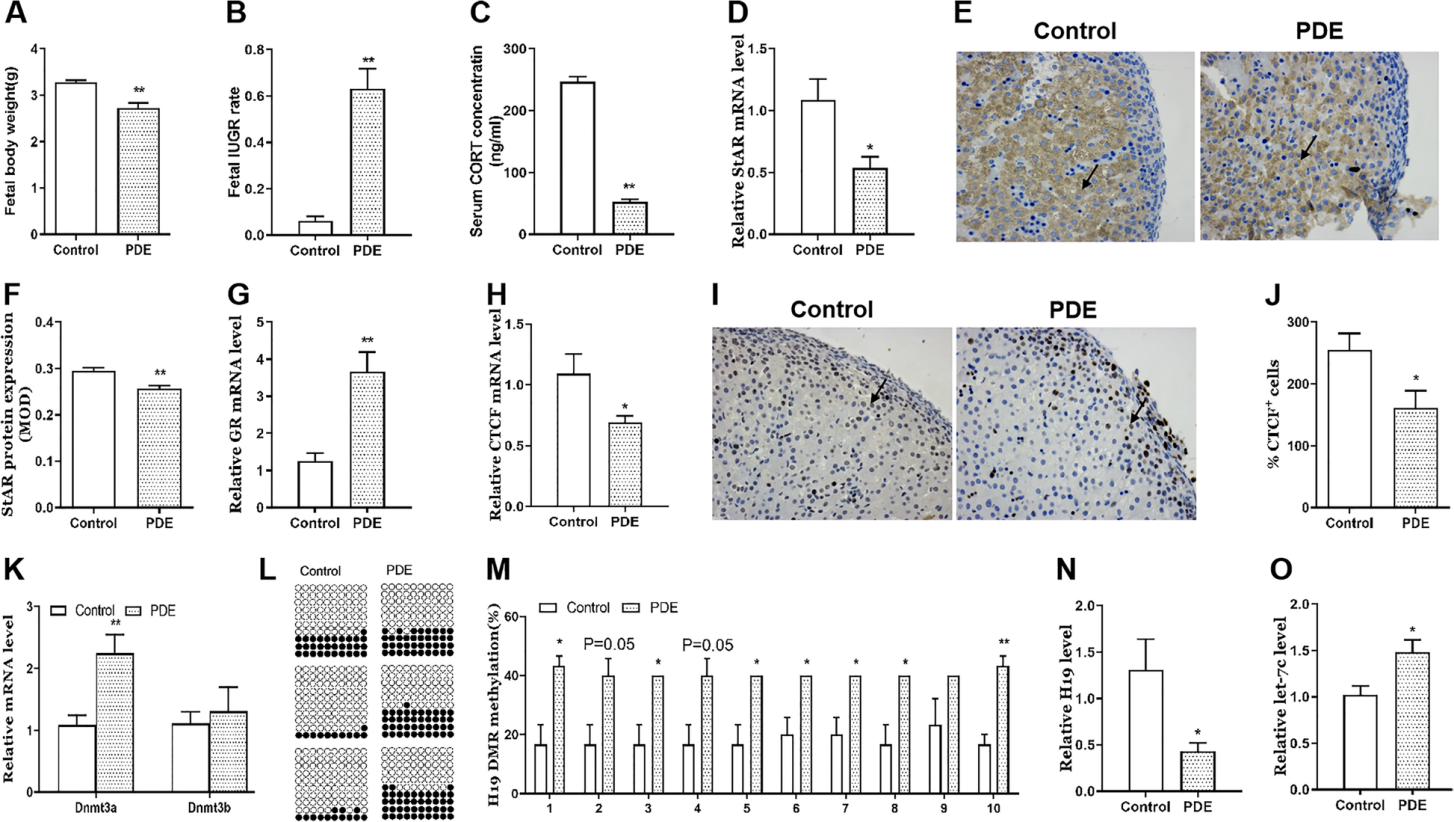
Effects of PDE on bodyweights, adrenal steroidogenesis and related H19/let-7c axis in female fetal rats. **A** bodyweights; **B** IUGR rate; **C** serum CORT concentration; **D** StAR mRNA expression; **E** **F** StAR protein expression (immunohistochemistry staining with brown, × 400); **G** GR mRNA expression; **H** CTCF mRNA expression; **I**-**J** CTCF protein expression (immunohistochemistry staining with brown, × 400); **K** Dnmt3a/3b mRNA expression; **L** **M** methylation status of individual DNA strands of H19 DMR containing 10 CpG sites and average methylation ratio in each CpG site; **N** H19 mRNA expression; **O** let-7c expression. Mean ± S.E.M., n = 10 for mRNA detection, *n* = 3 for immunohistochemistry detection, *n* = 6 for let-7c detection. ^*^p < 0.05, ^**^p < 0.01 *vs.* control. PDE, prenatal dexamethasone exposure; IUGR: intrauterine growth retardation; CORT, corticosterone; StAR, steroidogenic acute regulatory protein; GR, glucocorticoid receptor; CTCF, CCCTC binding factor; Dnmt3a/3b, DNA methyltransferase 3a/3b

Firstly, we observed the changes in bodyweights and adrenal steroidogenesis of fetal rats in our established PDE rat model (GD9-20, 0.2 mg/kg·d dexamethasone subcutaneously). Compared with the control group, bodyweights of PDE fetal rats were reduced (*p* < 0.01, Fig. 2A), and IUGR rates were increased (*p* < 0.01, Fig. 2B). Meanwhile, serum corticosterone levels were decreased (*p* < 0.01, Fig. 2C), and adrenal StAR mRNA and protein expression were significantly reduced in PDE group (*p* < 0.05, *p* < 0.01, Fig. 2D-F). Further, we explored the potential regulatory mechanism of the low steroidogenesis in PDE fetal rats. The results showed that PDE increased adrenal GR mRNA expression in fetal female rats (*p* < 0.01, Fig. 2G), decreased CTCF mRNA and protein expression (*p* < 0.05, Fig. 2H-J), and increased Dnmt3a expression (*p* < 0.01, Fig. 2K) while no change in Dnmt3b expression (Fig. 2K). Besides, methylation at multiple sites of H19 DMR in fetal rats were increased (*p* < 0.05, *p* < 0.01, Fig. 2L, M). At the same time, adrenal H19 expression was decreased (*p* < 0.05, Fig. 2N) and let-7c expression was increased (*p* < 0.05, Fig. 2O). These results suggested that PDE could inhibit adrenal steroidogenesis and its possible H19/let-7c axis regulation pathway in female fetal rats.

### Effects of dexamethasone on H19/let-7b axis and StAR expression in adrenocortical cells

**Fig. 3:**
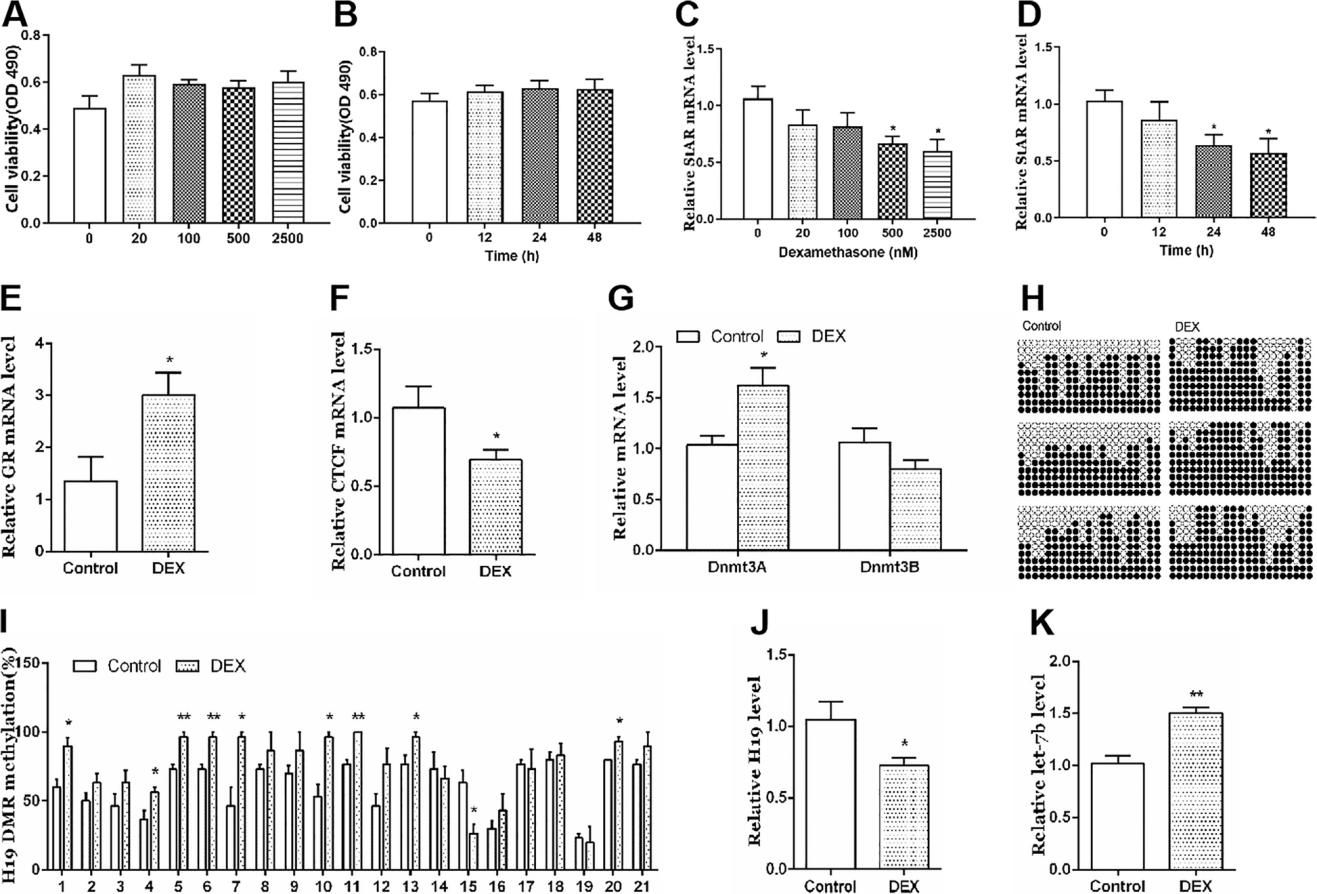
Effects of DEX on StAR expression and related H19/let-7b axis in NCI-H295R cells. **A** cell viability with 500 nM DEX treatment in different time; **B** cell viability with different concentrations of DEX treatment for 24 h; **C** StAR mRNA expression with DEX different concentrations; **D** StAR mRNA expression with DEX different time; **E** GR mRNA expression; **F** CTCF mRNA expression; **G** Dnmt3a and Dnmt3b mRNA expression; **H** **I** methylation status of individual DNA strands of H19 DMR containing 21 CpG sites and average methylation ratio in each CpG site; **J** H19 mRNA expression; **K** let-7b expression. Mean ± S.E.M., *n* = 6 for mRNA detection and *n* = 3 for BSP. ^*^p < 0.05, ^**^p < 0.01 *vs.* control. DEX, dexamethasone; StAR, steroidogenic acute regulatory protein; GR, glucocorticoid receptor; CTCF, CCCTC binding factor; Dnmt3a/3b, DNA methyltransferase 3a/3b

Next, we used a series of cell experiments to confirm the direct effect of dexamethasone on the StAR expression, and tested its possible molecular mechanisms in human adrenocortical cell line (NCI-H295R). Firstly, we treated the cells with dexamethasone at 500 nM for different time (12, 24 and 48 h) or at different concentrations (0, 20, 100, 500 and 2500 nM) for 24 h. The results showed that the cell viability did not significantly change (Fig. 3A, B), StAR expression was decreased in time- or concentration-dependent manners (*p* < 0.05, Fig. 3C, D). The above results showed that cell viability remained unchanged after treatment with dexamethasone concentrations (500 and 2500 nM) for 24 and 48 h, but the StAR expression was significantly reduced. The previous study suggested that the concentration of dexamethasone in fetal blood was 280 nM, so we selected 500 nM dexamethasone to treat cells for 24 h in the follow-up mechanism exploration experiment. Further, we treated the cells with 500 nM dexamethasone for 24 h, and found that GR mRNA expression was increased (*p* < 0.05, Fig. 3E), accompanied with the down-regulation of CTCF (*p* < 0.05, Fig. 3F) and up-regulation of Dnmt3a (*p* < 0.05, Fig. 3G). Meanwhile, the methylation levels at multiple sites of H19 DMR in the dexamethasone group significantly increased (*p* < 0.05, *p* < 0.01, Fig. 3H, I), and H19 expression was decreased (*p* < 0.05, Fig. 3J), while let-7b expression was increased (*p* < 0.01, Fig. 3K). These results suggested that dexamethasone could inhibit StAR expression and change the H19/let-7b axis-related pathway in the adrenocortical cells.

### H19/let-7b axis mediated dexamethasone effect on StAR expression in adrenocortical cells

**Fig. 4:**
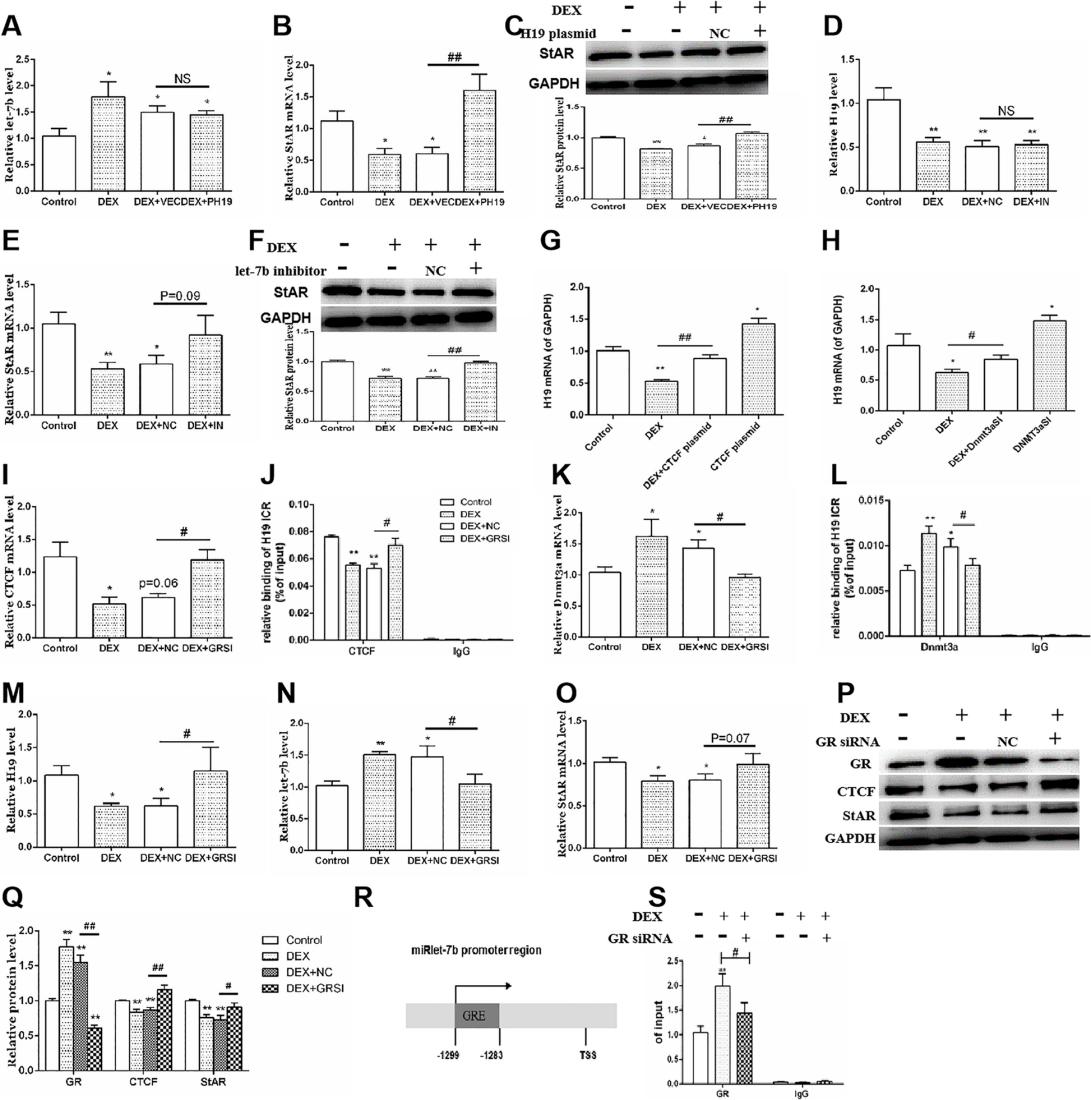
H19/let-7b axis mediated the effect of DEX on StAR expression in NCI-H295R cells. **A** Let-7b expression with H19 plasmid treatment; **B** StAR mRNA expression with H19 plasmid treatment; **C** StAR protein expression with H19 plasmid treatment; **D** H19 expression with let-7b inhibitor treatment; **E** StAR mRNA expression with let-7b inhibitor treatment; **F** StAR protein expression with let-7b inhibitor treatment; **G** H19 mRNA expression with CTCF plasmid treatment; **H** H19 mRNA expression with Dnmt3a siRNA treatment; **I** CTCF mRNA expression with GR siRNA treatment; **J** CTCF binding of H19 ICR with GR siRNA treatment; **K** Dnmt3a mRNA expression with GR siRNA treatment; **L** Dnmt3a binding of H19 ICR with GR siRNA treatment; **M** H19 mRNA expression with GR siRNA treatment; **N** Let-7b mRNA expression with GR siRNA treatment; **O** StAR mRNA expression with GR siRNA treatment; **P** **Q** GR, CTCF and StAR protein expression with GR siRNA treatment; **R** GRE in let-7b promoter region; **S** GR binding of let-7b. Mean ± S.E.M., *n* = 6 for RNA detection and *n* = 3 for western blot. ^*^p < 0.05, ^**^p < 0.01 *vs.* respective controls. DEX, dexamethasone; StAR, steroidogenic acute regulatory protein; CTCF, CCCTC binding factor; Dnmt3a, DNA methyltransferase 3a; GR, glucocorticoid receptor; GRE, glucocorticoid receptor element

To confirm the regulatory effects of H19/let-7b axis on the StAR expression, we intervened on H19 and let-7b, respectively. We found that H19 overexpression did not change let-7b expression (Fig. 4A) while reversed the inhibition of StAR mRNA and protein expression induced by dexamethasone (*p* < 0.05, *p* < 0.01, Fig. 4B, C). Similarly, the let-7b inhibitor did not change H19 expression while significantly reversed the inhibition of StAR mRNA and protein expression induced by dexamethasone (*p* < 0.01, Fig. 4E, F). It suggested that H19 and let-7b mediated the inhibition of StAR expression induced by dexamethasone in adrenocortical cells. Next, to confirm that CTCF/Dnmt3a mediated the change in H19 expression, we tested H19 expression in the presence of CTCF overexpression and Dnmt3a interference. Compared with the control group, CTCF overexpression and Dnmt3a siRNA significantly reversed the inhibitory effect of dexamethasone on H19 mRNA expression (*p* < 0.01, Fig. 4G, H). These results suggested that CTCF/Dnmt3a mediated the inhibition of H19 expression induced by dexamethasone in the adrenocortical cells.

Moreover, we treat adrenocortical cells with GR siRNA to confirm that GR mediated dexamethasone's effects. We found that GR siRNA could increase CTCF mRNA expression and its binding to the H19 ICR region (*p* < 0.05, *p* < 0.01, Fig. 4I, J), and decreased Dnmt3a mRNA expression and its binding to H19 ICR region (*p* < 0.05, *p* < 0.01, Fig. 4K, L). Meanwhile, GR interference could reverse the inhibition of H19 on StAR expression (*p* < 0.05, Fig. 4M, O, P and Q), and the promotion of let-7b expression on StAR expression (*p* < 0.05, Fig. 4N). Our bioinformatics analysis also found that there existed a binding site between GR and let-7b promoter region (-1283 ~ -1299, Fig. 4R). ChIP-PCR results confirmed that the binding level of GR to the let-7b promoter region significantly increased after dexamethasone treatment, while GR siRNA reversed this effect (*p* < 0.05, *p* < 0.01, Fig. 4S). In all, these results suggested that GR via CTCF/Dnmt3a mediated dexamethasone-induced H19/let-7b axis changes and StAR expression inhibition in human adrenocortical cells.

### Effects of PDE on adrenal steroidogenesis in offspring rats with matrilineal multigeneration inheritance and its H19/let-7c axis transmission mechanism

#### Effects of PDE on adrenal H19/let-7c axis and steroidogenesis in the F1 female offspring rats

**Fig. 5:**
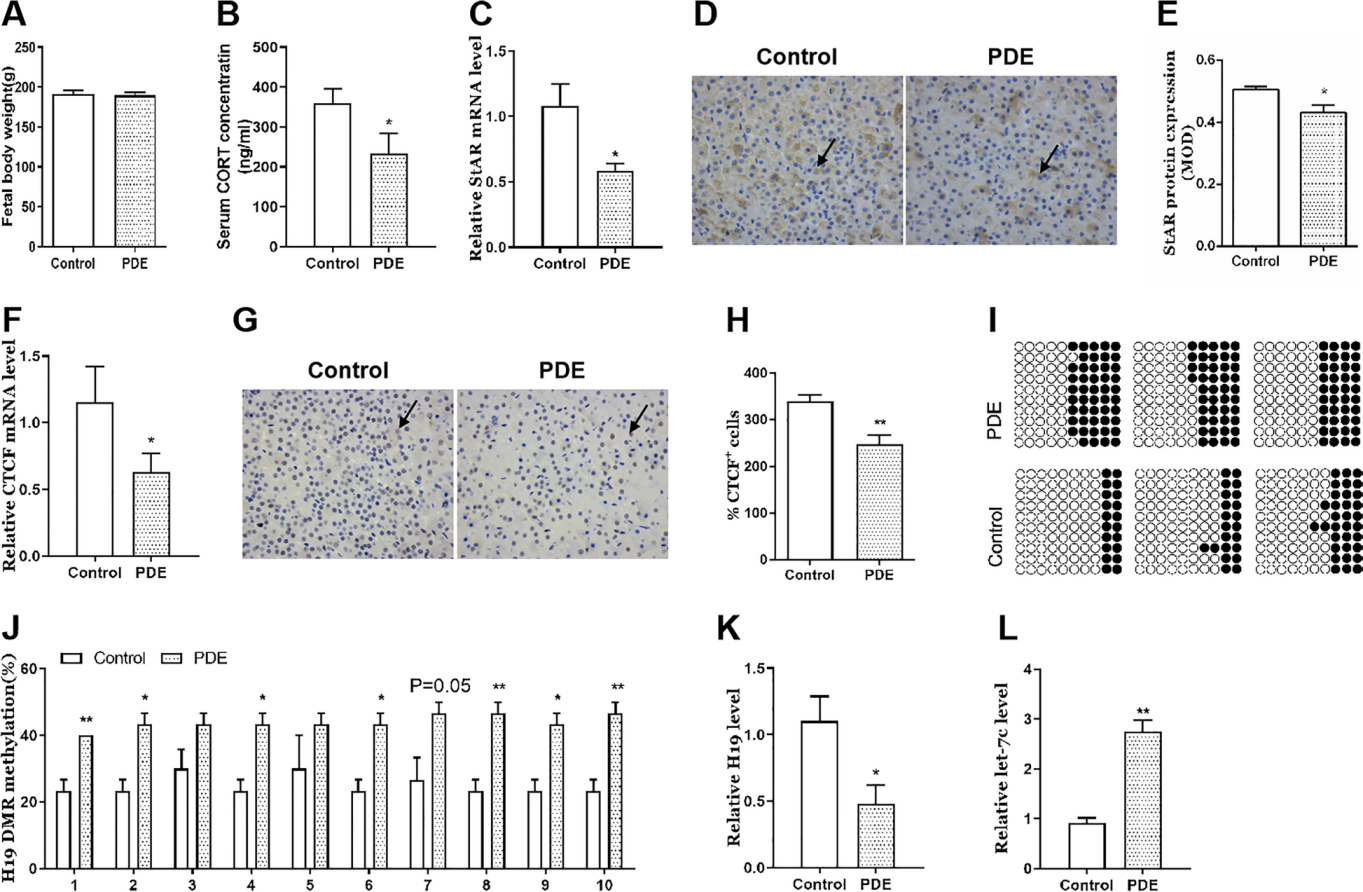
Effects of PDE on adrenal H19/let-7c axis and steroidogenesis in female offspring rats of F1 generation. **A** Bodyweight; **B** Serum CORT concentration; **C** Adrenal StAR mRNA expression; **D** **E** Adrenal StAR protein expression; **F** Adrenal CTCF mRNA expression; **G** **H** Adrenal CTCF protein expression (immunohistochemistry staining with brown, × 400); **I** **J** Adrenal methylation status of individual DNA strands of H19 DMR containing 10 CpG sites and average methylation ratio in each CpG site; **K** Adrenal H19 mRNA expression; **L** Adrenal let-7c expression. Mean ± S.E.M., *n* = 12 for mRNA detection, *n* = 3 for immunohistochemistry detection and BSP. ^*^p < 0.05, ^**^p < 0.01 *vs*. control. PDE, prenatal dexamethasone exposure; CORT, corticosterone; StAR, steroidogenic acute regulatory protein; CTCF, CCCTC binding factor

We tested the changes in adrenal H19/let-7c axis and StAR expression of F1 female rats to explore if fetal adrenal toxicity could continue until after birth. We found there were no significant changes in bodyweight of PDE offspring rats (Fig. 5A), but serum CORT concentrations and adrenal StAR mRNA expression were both decreased compared with the control group (*p* < 0.05, Fig. 5B, C). Immunohistochemical results indicated that StAR protein expression was also significantly decreased (*p* < 0.05, Fig. 5D, E). Further, we observed the mechanism related indicators, and found adrenal CTCF mRNA and protein expression were decreased in PDE offspring rats (*p* < 0.05, Fig. 5F). Immunohistochemical results indicated that CTCF protein expression was also significantly decreased (*p* < 0.01, Fig. 5G, H). Meanwhile, the methylation levels of H19 DMR at multiple sites were increased in PDE group (*p* < 0.05, *p* < 0.01, Fig. 5I, J), and H19 expression was decreased (*p* < 0.05, Fig. 5K) while let-7c expression was increased (*p* < 0.01, Fig. 5L). These results suggested that PDE could inhibit the adrenal steroidogenesis, accompanied by the changes in H19/let-7c axis-related pathway after birth in female offspring rats of F1 generation.

### Effects of PDE on adrenal H19/let-7c axis and steroidogenesis in the F2 female offspring rats

**Fig. 6:**
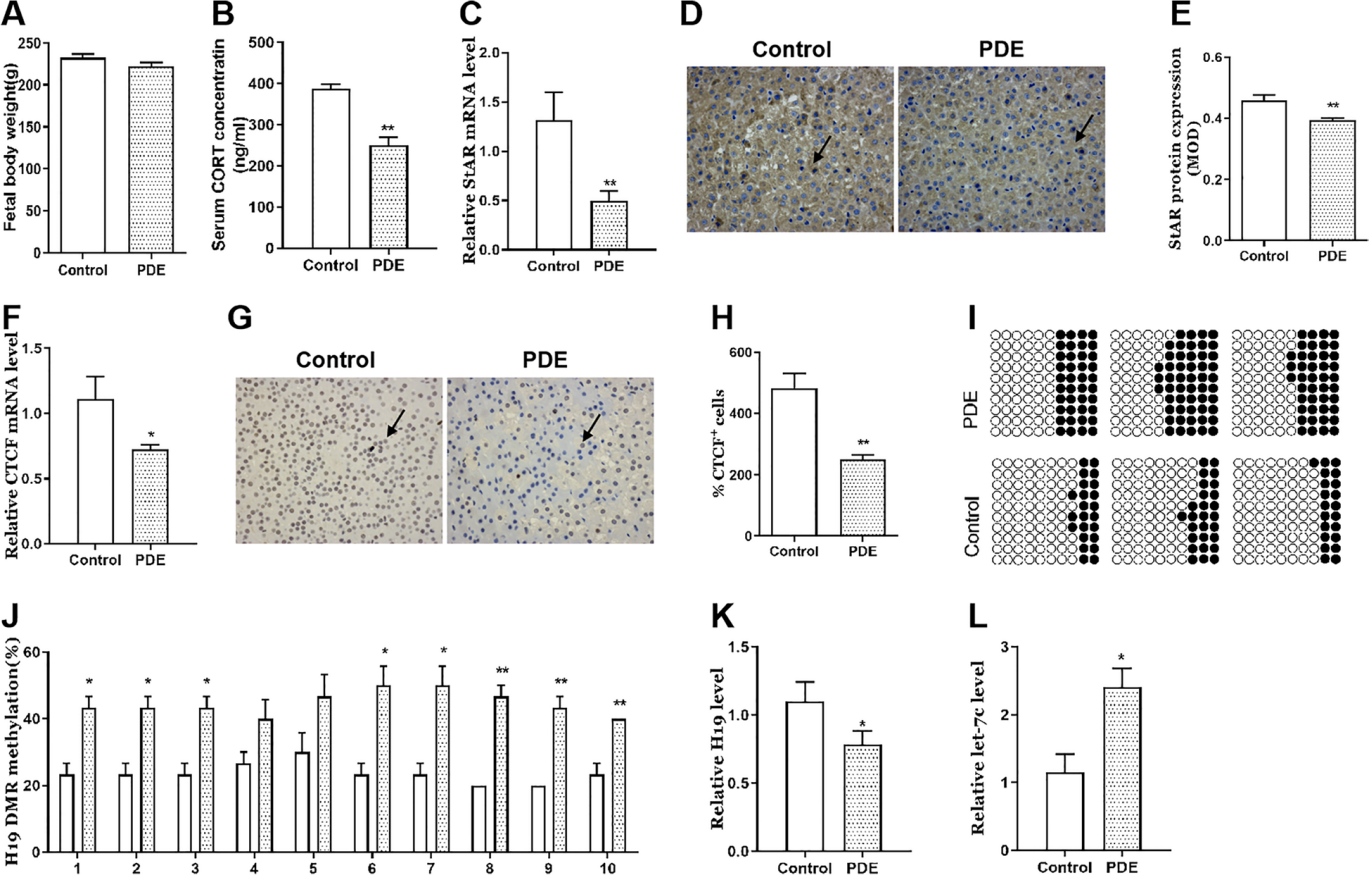
Effects of PDE on adrenal H19/let-7c axis and steroidogenesis in female offspring rats of F2 generation **A** Bodyweight; **B** Serum CORT concentration; **C** Adrenal StAR mRNA expression; **D** **E**)Adrenal StAR protein expression; **F** Adrenal CTCF mRNA expression; **G** **H** Adrenal CTCF protein expression (immunohistochemistry staining with brown, × 400); **I** **J** Adrenal methylation status of individual DNA strands of H19 DMR containing 10 CpG sites and average methylation ratio in each CpG site; **K** Adrenal H19 mRNA expression; **L** Adrenal let-7c expression. Mean ± S.E.M., *n* = 12 for mRNA detection, *n* = 3 for immunohistochemistry detection and BSP. ^*^p < 0.05, ^**^p < 0.01 *vs*. control. PDE, prenatal dexamethasone exposure; CORT, corticosterone; StAR, steroidogenic acute regulatory protein; CTCF, CCCTC binding factor

To explore whether the adrenal toxicity of PDE could continue to the F2 (maternal inheritance), we tested the changes in adrenal H19/let-7c axis and steroidogenesis of F2 female rats in PW8. We found there were no significant change in bodyweight of PDE offspring rats (Fig. 6A), but serum CORT concentration and adrenal StAR expression were both decreased compared with the control group (*p* < 0.01, Fig. 6B-E). Furthermore, adrenal CTCF mRNA and protein expression were decreased in PDE group (*p* < 0.05, *p* < 0.01, Fig. 6F-H). Meanwhile, the methylation levels of H19 DMR at multiple sites were increased in PDE group (*p* < 0.05, *p* < 0.01, Fig. 6I, J), and H19 expression was decreased (*p* < 0.05, Fig. 6K) while let-7c expression was increased (*p* < 0.05, Fig. 6L). These results suggested that PDE could inhibit the adrenal steroidogenesis, accompanied by the changes in H19/let-7c axis-related pathway after birth in female offspring rats of F2 generation, which were consistent with that of the female F1 generation.

### Effects of PDE on adrenal H19/let-7c axis and steroidogenesis in the F3 female offspring rats

**Fig. 7:**
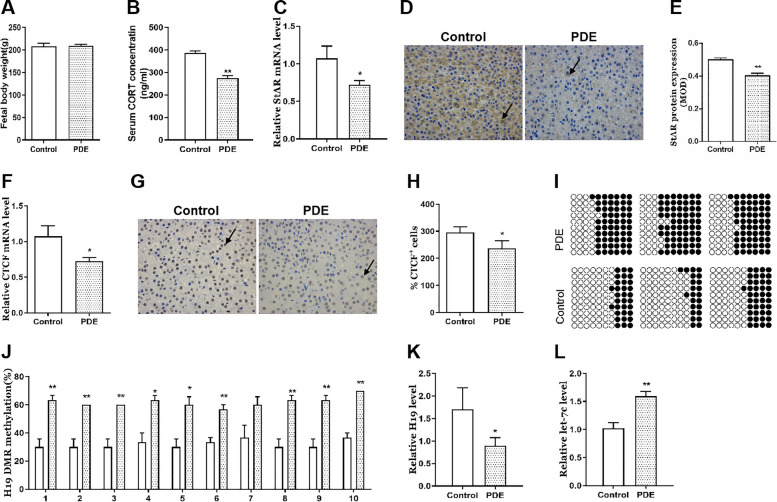
Effects of PDE on adrenal H19/let-7c axis and steroidogenesis in female offspring rats of F3 generation **A** Bodyweight; **B** Serum CORT concentration; **C** Adrenal StAR mRNA expression; **D** **E** Adrenal StAR protein expression; **F** Adrenal CTCF mRNA expression; **G** **H** Adrenal CTCF protein expression (immunohistochemistry staining with brown, × 400); **I** **J** Adrenal methylation status of individual DNA strands of H19 DMR containing 10 CpG sites and average methylation ratio in each CpG site; **K** Adrenal H19 mRNA expression; **L** Adrenal let-7c expression. Mean ± S.E.M., *n* = 12 for mRNA detection, *n* = 3 for immunohistochemistry detection and BSP. ^*^p < 0.05, ^**^p < 0.01 *vs*. control. PDE, prenatal dexamethasone exposure; CORT, corticosterone; StAR, steroidogenic acute regulatory protein; CTCF, CCCTC binding factor

To investigate whether PDE-induced adrenal development toxicity could be extended to the F3 (maternal multigenerational inheritance), we tested the changes in adrenal H19/let-7c axis and steroidogenesis of F3 female rats in PW8. We found there were no significant changes in bodyweight of PDE offspring rats (Fig. 7A), but serum CORT concentrations and adrenal StAR expression were both decreased compared with the control group (*p* < 0.05, *p* < 0.01, Fig. 7B-E). Further, we observed the mechanism related indicators, and found adrenal CTCF mRNA and protein expression were decreased in the PDE group (*p* < 0.05, Fig. 7F-H). Meanwhile, the methylation levels of H19 DMR at multiple sites were increased in the PDE group (*p* < 0.05, *p* < 0.01, Fig. 7I, J), and H19 expression was decreased (*p* < 0.05, Fig. 7K) while let-7c expression was increased (*p* < 0.01, Fig. 7L). These results suggested that PDE could inhibit the adrenal steroidogenesis, accompanied by the changes in H19/let-7c axis related pathway after birth in female offspring rats of F3 generation, which were consistent with that of the female F1 and F2 generation.

### Effects of PDE on H19 and let-7c expression in oocytes of F2 and F3 generations

**Fig. 8:**
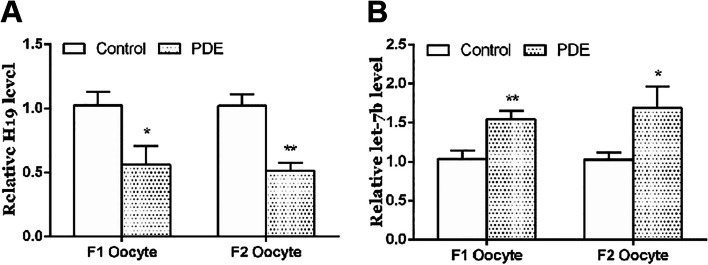
Effects of PDE on H19 and let-7c expression in oocytes of F2 and F3 generations. **A** H19 mRNA expression; **B** let-7c expression. Mean ± S.E.M., *n* = 12 for mRNA detection, ^*^p < 0.05, ^**^p < 0.01 *vs.* control. PDE, prenatal dexamethasone exposure

To confirm that the inhibition of adrenal steroidogenesis caused by PDE was mediated by germ cells, we tested the oocytes in PDE rats. PMSG and HCG were intraperitoneally injected for superovulation treatment in F1 and F2 adult female rats. After that, ovaries were removed and treated with hyaluronidase to isolate oocytes, then detect the expression of H19 and let-7c in oocytes. We found that compared with control group, the expression of H19 in PDE oocytes of the F1 and F2 was decreased (*p* < 0.05, *p* < 0.01, Fig. 8A), while the expression of let-7c was increased (*p* < 0.05, *p* < 0.01, Fig. 8B). It suggested that the multigenerational inheritance of PDE-induced low adrenal steroidogenesis might be caused by the down-regulation of H19 and upregulation of let-7c in oocytes.

## Discussions

### PDE inhibits adrenal steroidogenesis in female offspring rats and has a maternal multigenerational inheritance effect

During the development period, the fetus was most sensitive to endocrine disruptors and likely to adapt to the environment by reacting to adverse environmental conditions. But when the offspring are freed from the hostile environment, the initial adaptive change may evolve into an “over-adaptive” state of disease susceptibility [37]. As an important endocrine organ, the adrenal gland is highly susceptible to endocrine disruptors. Clinical studies have shown that dexamethasone administration during middle and late stages of pregnancy could improve the outcome of premature infants [38], so dexamethasone was widely used to treat pregnant women who were at risk of preterm delivery at 24–34 weeks (second and third trimesters). Adrenocortical functional differentiation began from GD9, so we choose to administered dexamethasone in GD9-20, which is equivalent to the second and third trimesters of pregnancy. In addition, the National Institutes of Health (NIH) recommends that the prenatal dose of dexamethasone for preterm labor treatment is 6 mg intramuscular every 12 h (4 cycles per course) [39, 40]. Based on the dose conversion between humans and rats (1:6.17) [41], the mentioned dexamethasone dose above for clinical pregnant women is equivalent to 0.31 mg/kg·d administered to rats, so the dexamethasone dose (0.2 mg/kg·d) used was lower than the clinical dose in this study. In conclusion, based on the PDE rat model established in this study, the study on transgenerational inheritance of adrenal developmental toxicity and its mechanism is of great significance for guiding clinical rational drug use and exploring early intervention targets.

In our previous studies, we confirmed that PDE could inhibit the adrenal steroidogenesis in male offspring through regulating the H3K27ac level of SF1 promoter region and its expression [42]. However, the intergenerational inheritance of adrenal developmental toxicity induced by PDE has not been reported. In this study, we confirmed that adrenal steroidogenic function (including blood corticosterone concentration and adrenal StAR expression) is inhibited in the female PDE offspring rats before and after birth. Furthermore, the adrenal steroidogenic function is still inhibited in the F2 and F3 female PDE offspring generation. Similar phenomena also appeared in the male adrenal steroidogenic function of F1, F2 and F3 generations after birth (Fig. S1). However, the decline degree in the F1 females is more obvious than the F1 males, and the sex difference faded away in the F2/F3 females and males. In conclusion, the inhibition of F1 adrenal steroidogenesis induced by PDE can be inherited maternally to F2 and F3 generations, suggesting the existence of multigenerational inheritance effect (including trans-generational inheritance).

### GR via CTCF/Dnmt3a mediated dexamethasone-induced StAR-related-H19/let-7b axis changes

More and more studies found that abnormal fetal epigenetic modification caused by adverse environment exposure during pregnancy played a decisive role in postnatal multi-organ development programming and functional homeostasis [43, 44]. Dexamethasone easily passes through placenta into the fetal circulation and regulates a series of downstream functional genes through GR. It has been reported that miRNA let-7 family could significantly affect StAR expression in granular cells [36]. In this study, bioinformatics analysis found a binding site between GR and let-7b promoter region (-1283 ~ -1299). Combined with in vivo and in vitro experiments, we confirmed that the activated GR by dexamethasone could bind to the let-7b promoter region to increase let-7b expression, which reducing StAR expression and eventually inhibiting adrenal steroidogenesis.

As a maternal imprinted gene, H19 can transmit maternally imprinting state phenotype to offspring. A study has shown a “molecular sponge” effect between imprinted gene H19 and let-7, and H19 can significantly change the impact of miRNA let-7 on target gene expression [31, 45]. Therefore, we further explored the role of H19/Let-7 axis-related pathways in regulating adrenal steroidogenesis in PDE offspring rats. In this study, we found that PDE reduced the expression of fetal adrenal H19, and dexamethasone in vitro also could inhibit the expression of H19. Besides, H19 overexpression could reverse the inhibition of StAR expression induced by dexamethasone without affecting let-7b expression, and the let-7b inhibitor also can’t alter the effect of dexamethasone on H19 expression. These results suggested that although there is no interaction between H19 and let-7, the “molecular sponge” effect between H19 and let-7 could regulate the inhibitory effect of let-7 on the StAR expression.

**Fig. 9:**
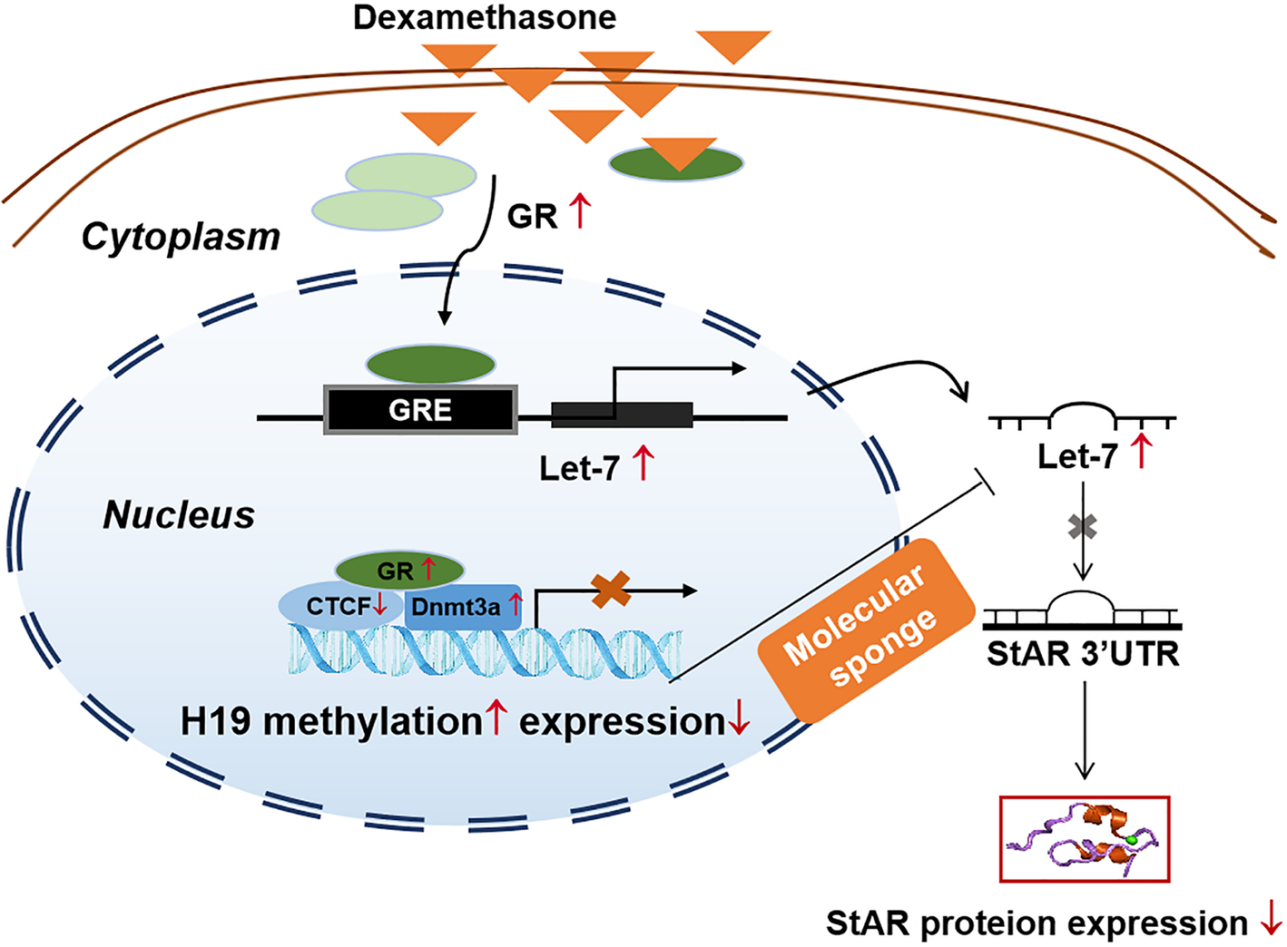
H19/let-7 axis mediated inhibitory effect of dexamethasone on StAR expression in adrenocortical cells. GR, glucocorticoid receptor; CTCF, CCCTC binding factor; Dnmt3a, DNA methyltransferase 3 alpha; StAR, steroidogenic acute regulatory protein

Studies have shown that CTCF could specifically combine with H19 DMR to change its epigenetic modification (such as DNA methylation), adjusting the H19 imprinting formations [46, 47]. In the process of CTCF regulating gene imprinting of H19, DNMTs (including DNMT1 and DNMT3a/b) is involved in transcriptional regulation of H9 by competing for binding sites of CTCF in H19 DMR region [29]. In this study, combined with the results of in vivo and in vitro experiments, and a series of interference experiments (*e.g.* GR siRNA, Dnmt3a siRNA and CTCF plasmid) in vitro, we found that dexamethasone, through the activation of GR, on the one hand, could decrease CTCF expression and its binding to H19 DMR region, on the other hand recruited Dnmt3a and increased its binding to H19, resulting in hypermethylation and low expression of H19. The low expression of H19 can enhance the inhibitory effect of let-7 on StAR expression by weakening the "molecular sponge" effect between H19 and let-7 in adrenal cortical cells (Fig. 9).

### PDE-induced inhibition of maternal multigenerational adrenal steroidogenesis was transmitted in oocytes via H19/let-7 axis

More and more studies have shown that epigenetic programming changes caused by adverse environments during pregnancy can continue after birth, and the traits of the parents can be passed on to the offspring for multiple generations in a normal living environment [48, 49]. Our previous researches also found that the developmental programming of multiple organs (such as testes, ovaries, and long bones) in PDE progeny rats was altered, and they were susceptible to related diseases after birth, which continued to adulthood of the F2 or the F3 [12, 13, 15]. In this study, adrenal steroidogenesis in female and male PDE offspring rats of F2 generation were reduced, which was consistent with the results of F1 generation. It suggested that the inhibition of adrenal steroidogenesis in PDE offspring rats can be extended through maternal line to puberty in F2 generation, which existed an “intergenerational inheritance” effect. “Transgenerational inheritance” means the phenomena that could not be ascribed to the direct effects of a particular trigger on the affected organism. It is well known that adverse environmental factors during pregnancy can directly affect the embryo (F1) or oocyte (F2) already formed in female embryo [50]. Therefore, only maternal traits that occurred in the third generation of female offspring (F3, inherited from the maternal line) can be defined as a “transgenerational inheritance”. In this study, dexamethasone had no direct effect on the F3 female offspring rats in the PDE group, but adrenal steroidogenesis was still reduced after birth, which was consistent with the changes in the F1 and F2 female offspring rats. These results suggest that the inhibition of adrenal steroidogenesis in offspring by PDE has a transgenerational inheritance effect.

Parental dependence in acquired phenotypes strongly suggests that epigenetic markers transmitted by germ cells may be biological foundation for multigenerational inheritance [51]. Epigenetic information could be tracked in germ cells and transmitted to offspring [26, 52]. We obtained same results on the epigenetic information of imprinted gene H19 in PDE female oocytes of the F1 and F2 generation, and confirmed that the epigenetic information carried by H19 escaped the reprogramming effect of oocyte formation and early embryonic development, which inheriting to offspring (including the F2 and F3 generation). Meanwhile, we found that let-7c expression in female PDE oocytes of the F1 and F2 generations were increased. Studies have shown that miRNA in germ cells can be transmitted to offspring and cause generational inheritance of corresponding traits [53]. Hence, we proposed that the epigenetic information carried by H19/let-7c axis was transmitted by oocytes, which could mediate the inhibition of adrenal steroidogenesis in PDE offspring rats of the F2 and F3 generations.

Interestingly, we have confirmed that prenatal caffeine exposure (PCE) can also induce adrenal dysfunction in F1, F2, and F3 female offspring rats with maternal multigenerational inheritance. Surprisingly, but the F1 and F3 generation rats with PCE showed decreased adrenal steroidogenesis, while the F2 generation rats showed increased adrenal steroidogenesis [54]. Further, we confirmed that the differential changes in adrenal steroidogenesis of three generations were related to changes in developmental programming induced by intrauterine over-exposure or low-exposure to maternal glucocorticoids [54]. Although the related molecular mechanisms regulating altered StAR expression was very similar, this “contemporary endogenous glucocorticoid-induced programming alteration and its transgenerational inheritance effect” in PCE rat model is significantly different from the dexamethasone-induced programming alteration and its transgenerational inheritance effect of F0 generation in PDE rat model. We suggest that the phenotypic difference between PCE and PDE rat models is due to different inducement (endogenous corticosterone or exogenous dexamethasone) exposed to fetal rats in utero of F1 generation. The transgenerational inheritance effects means that modifications acquired in the F0 generation would need to be observed in the F1 and F2 generations, and potentially beyond. For pregnant females that experience environmentally-induced epigenetic changes in this study, the intergenerational effects encompass transmission from F0 to F2, and only persistence to the F3 generation would be considered as transgenerational inheritance. So, the PDE induced adrenal dysfunction is transgenerational inheritance other than the PCE (called intergenerational inheritance).

### Sex difference in multigenerational inheritance mechanism of adrenal steroidogenesis inhibition induced by PDE

Studies suggested that gender difference in the growth and development of offspring under adverse pregnancy conditions begin in the intrauterine period and may have long-term effects [55–57]. Different subtypes of GR mediate the difference in glucocorticoid sensitivity, which may be an important reason for the gender difference in fetal developmental toxicity and long-term disease susceptibility in offspring [58]. It was found that multiple subtypes generated by selective splicing of GR transcription and different translation initiation sites were important reasons for the difference in glucocorticoid sensitivity [59]. For example, there are gender differences in placental GR subtype expression and its sensitivity to glucocorticoids, resulting in gender differences in fetal developmental toxicity and long-term health [58]. In this study, we found the adrenal let-7c was increased in the PDE male offpring of F1 generation and no signigicant change in F2 and F3 generation while decreased in the PDE females of F1, F2 and F3 generaion, which shows obvious sex differences PDE offspring rats (Fig. S2). The expression of let-7c was directly regulated by dexamethasone/GR. Therefore, we hypothesized that sex differences in let-7c expression might be caused by the sensitivity of adrenal GR to dexamethasone in fetal rats of different sexes. In addition to the direct regulation of StAR expression by GR/let-7 signaling, the methylation level and expression of imprinted gene H19 were involved in the regulation of StAR expression. In this study, the adrenal hypermethylation and low expression of H19 regulated by CTCF/Dnmt3a pathway of PDE offspring showed consistent changes in adrenal steroidogenesis of F1, F2 and F3 generations, without significant sex difference. These results suggest that the sex difference in adrenal steroidogenesis inhibition induced by PDE is mainly due to the difference in the GR/let-7 regulatory mechanism, and does not depend on the GR/CTCF/Dnmt3a/H19 regulatory mechanism.

In the follow-up study, we will conduct early integrated prevention and treatment studies based on the discovered trans-generational genetic, molecular targets of adrenal developmental toxicity caused by PDE, which includes building screening systems in vitro, early warning and drug prevention technologies. In recent years, our research team has done some work in this respect. For example, the changes of SF1-H3K27ac levels in neonatal peripheral blood mononuclear cells were detected to warn the adrenal hypofunction and related disease susceptibility in PDE progenies [42]. The changes of TGFβR1-H3K9ac levels in the human umbilical cord were detected to warn the susceptibility to chondrodysplasia/osteoarthritis in PDE progenies [60]. Administration of TGFβR1 agonist glucosamine can effectively prevent and treat osteoarthritis in PDE progeny [61]. Secondly, we will directly manipulate the expression of H19 and let-7b in oocytes (germ cells) through CRISPR/Cas9 technology, to confirm the H19/ let-7b-mediated transgenerational inheritance mechanism at the overall level.

## Conclusions

**Fig. 10:**
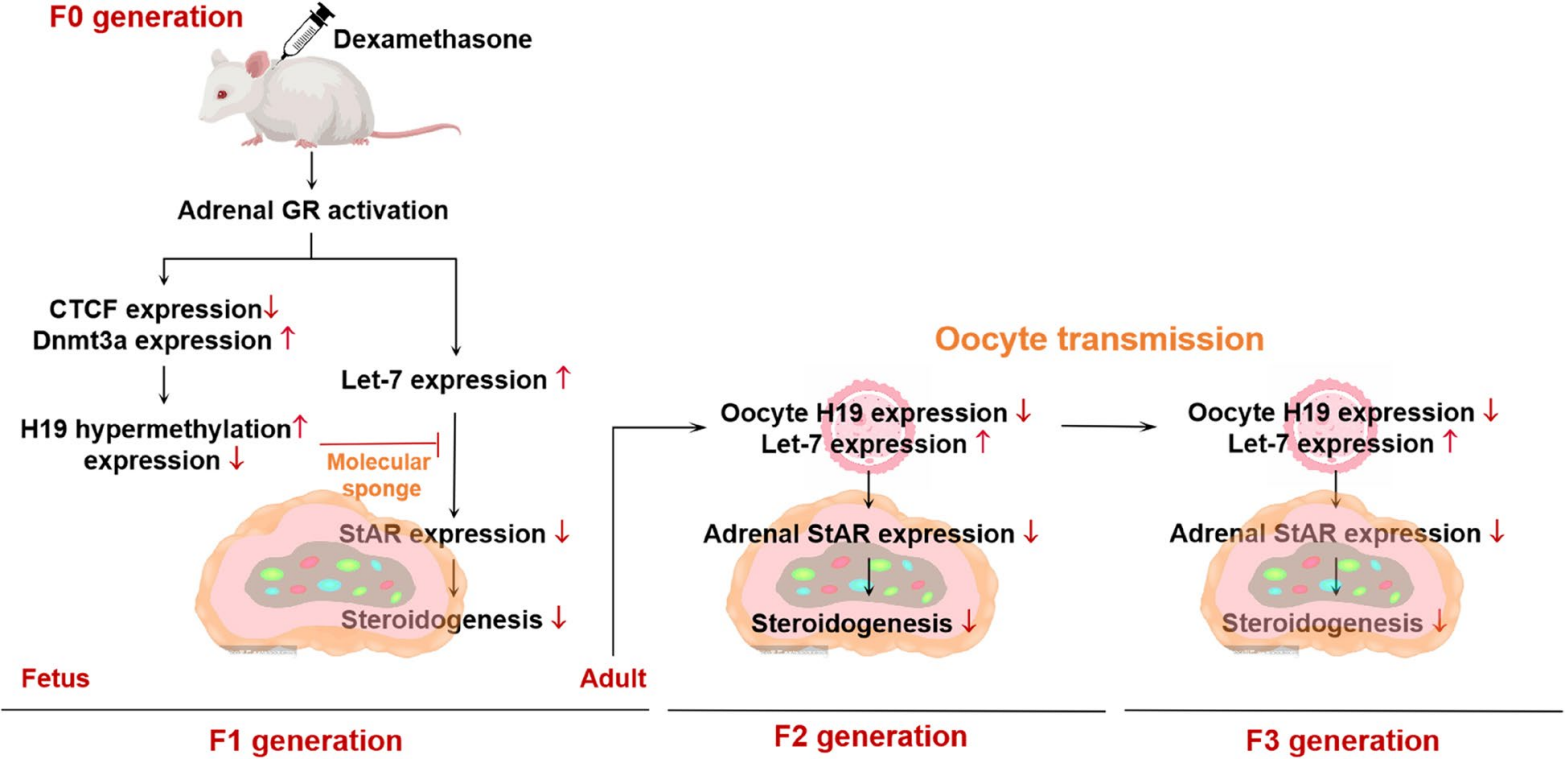
Intrauterine programming mechanism of transgenerational inheritance effects of adrenal steroidogenesis in female offspring rats induced by prenatal dexamethasone exposure. GR, glucocorticoid receptor; CTCF, CCCTC binding factor; Dnmt3a, DNA methyltransferase 3 alpha; StAR, steroidogenic acute regulatory protein

This study confirm that PDE could inhibit adrenal steroidogenesis in offspring through H19/let-7 axis, which existed a multigenerational inheritance effect by maternal line (Fig. 10). We confirmed that intrauterine dexamethasone exposure could activate fetal adrenal GR. On the one hand, GR reduced CTCF expression and its binding to H19 DMR region; on the other hand, it recruited Dnmt3a and increased its binding to H19, resulting in hypermethylation and low expression of H19. The low expression of H19 can enhance the inhibitory effect of let-7 on StAR expression by weakening the “molecular sponge” effect between H19 and let-7. The epigenetic information carried by H19/let-7c axis was transmitted by oocytes. Due to its genetic stability, F2 generation oocytes indirectly exposed to dexamethasone also showed the down-regulation of H19 and up-regulation of let-7c, which could be inherited to F3 generation. The cascade effect of CTCF/H19/Let-7b finally resulted in the transgenerational inheritance effect of adrenal steroidogenesis inhibition in PDE offspring. In addition, there is a sex difference in adrenal let-7c expression, suggested the sensitivity of let-7 to GR may determine the sex differences in adrenal steroidogenic function in the PDE offspring rats. This study provides a deep understanding of the intrauterine origin of adrenal developmental toxicity, presents experimental evidence for the transgenerational inheritance of acquired traits induced by PDE, and also provides theoretical basis for the exploration of the prevention and treatment targets of PDE-induced adrenal developmental toxicity and related fetal-original diseases in offspring.

### Supplementary Information

**Additional file 1:** **Fig S1. **Effects of PDE on adrenal steroidogenesis in male offspring rats of three generation. (A, D, and J) Adrenal CORT concentration; (B, E, H and K) Adrenal StAR mRNA expression; (C, F, I and L) Adrenal StAR protein expression. Mean ± S.E.M., *n*=12 for mRNA detection, *n*=3 for immunohistochemistry detection. ^*^p<0.05,^**^p<0.01*vs*. control. PDE, prenatal dexamethasone exposure; CORT, corticosterone; StAR, steroidogenic acute regulatory protein. **Fig S2. **Effects of PDE on adrenal let-7c expression in male offspring rats of three generation. (A) Fetal adrenal let-7c expression in F1 generation; (B) Adult adrenal let-7c expression in F1 generation; (C) Adult adrenal let-7c expression in F2 generation; (D) Adult adrenal let-7c expression in F3 generation;. Mean ± S.E.M., n=12 for mRNA detection. ^*^p<0.05, ^**^p<0.01*vs*. control. PDE, prenatal dexamethasone exposure.

## Data Availability

Data associated with the current study are available from the corresponding authors upon reasonable request.
