# Supplementary material for: Transgenerational inheritance of adrenal steroidogenesis inhibition induced by prenatal dexamethasone exposure and its intrauterine mechanism
Source: Cell Commun Signal. 2023 Oct 18;21:294. doi: 10.1186/s12964-023-01303-0 (PMC10585925; doi:10.1186/s12964-023-01303-0)
Supplement: Supplementary file 2 — Additional file 1: Fig S1. Effects of PDE on adrenal steroidogenesis in male offspring rats of three generation. (A, D, and J) Adrenal CORT concentration; (B, E, H and K) Adrenal StAR mRNA expression; (C, F, I and L) Adrenal StAR protein expression. Mean ± S.E.M., n=12 for mRNA detection, n=3 for immunohistochemistry detection. *p<0.05,**p<0.01vs. control. PDE, prenatal dexamethasone exposure; CORT, corticosterone; StAR, steroidogenic acute regulatory protein. Fig S2. Effects of PDE on adrenal let-7c expression in male offspring rats of three generation. (A) Fetal adrenal let-7c expression in F1 generation; (B) Adult adrenal let-7c expression in F1 generation; (C) Adult adrenal let-7c expression in F2 generation; (D) Adult adrenal let-7c expression in F3 generation;. Mean ± S.E.M., n=12 for mRNA detection. *p<0.05, **p<0.01vs. control. PDE, prenatal dexamethasone exposure. [file 12964_2023_1303_MOESM1_ESM.docx]

**Supplemental material**


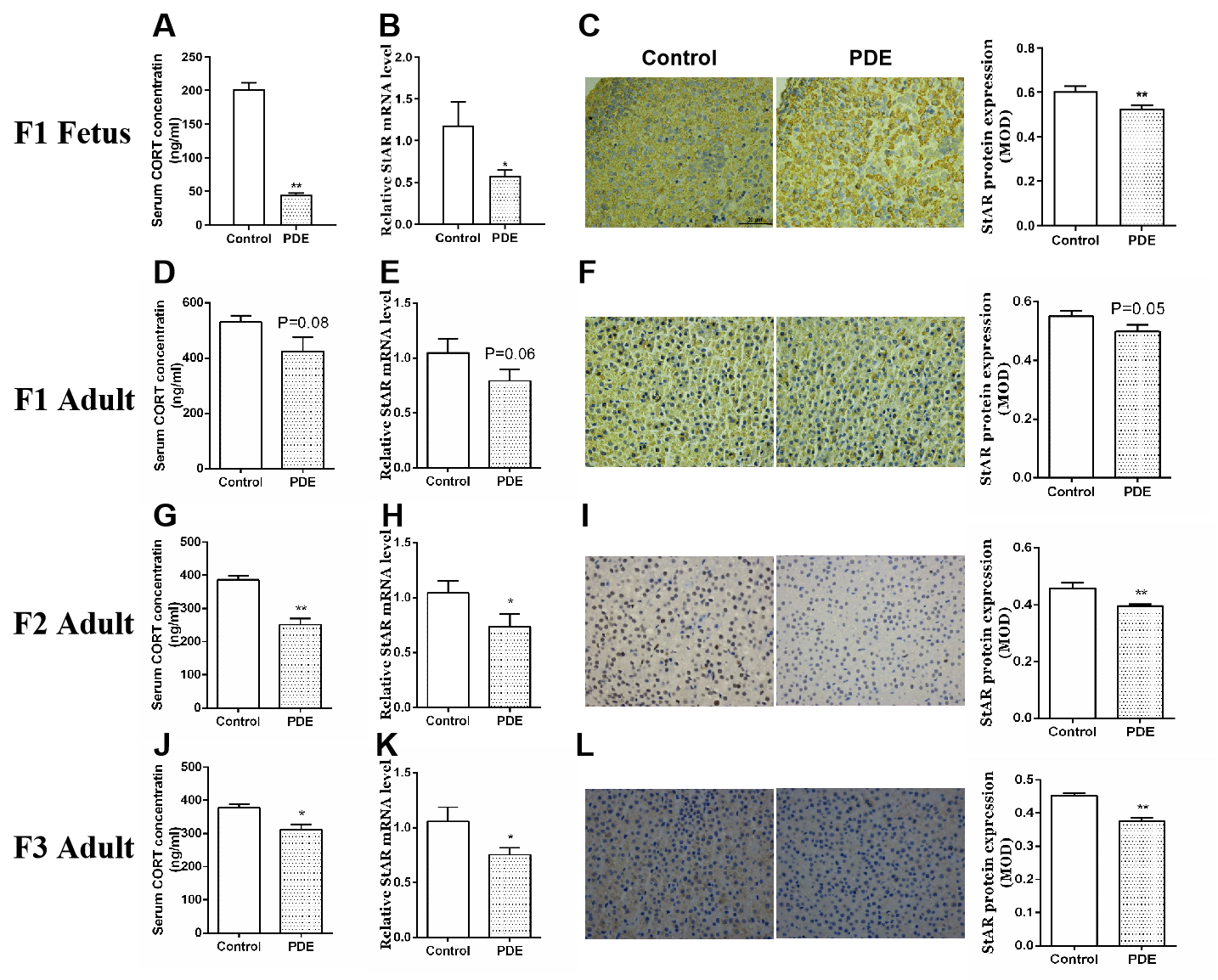


**Fig S1. Effects of PDE on adrenal steroidogenesis in male offspring rats of three generation.** (A, D, and J) Adrenal CORT concentration; (B, E, H and K) Adrenal StAR mRNA expression; (C, F, I and L) Adrenal StAR protein expression. Mean ± S.E.M., n=12 for mRNA detection, n=3 for immunohistochemistry detection. ^*^*p*<0.05, ^**^*p*<0.01 *vs*. control. PDE, prenatal dexamethasone exposure; CORT, corticosterone; StAR, steroidogenic acute regulatory protein.


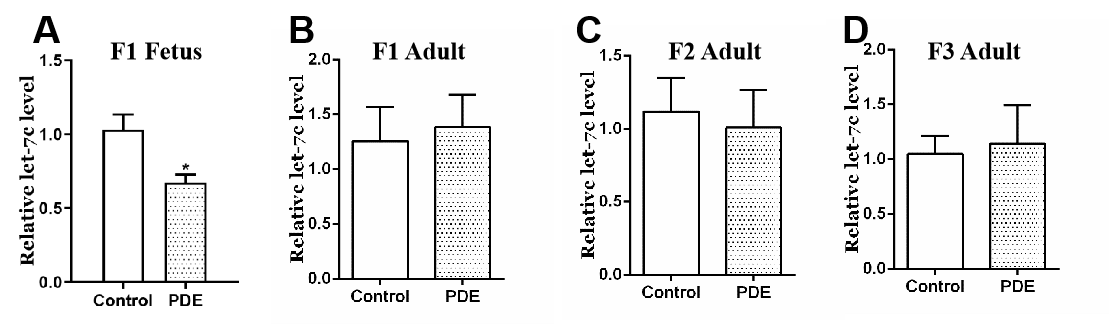


**Fig S2. Effects of PDE on adrenal let-7c expression in male offspring rats of three generation.** (A) Fetal adrenal let-7c expression in F1 generation; (B) Adult adrenal let-7c expression in F1 generation; (C) Adult adrenal let-7c expression in F2 generation; (D) Adult adrenal let-7c expression in F3 generation;. Mean ± S.E.M., n=12 for mRNA detection. ^*^*p*<0.05, ^**^*p*<0.01 *vs*. control. PDE, prenatal dexamethasone exposure.
